# The effect of comprehensive psychological interventions on the mental health of the community elderly

**DOI:** 10.3389/fpsyt.2024.1431116

**Published:** 2024-08-30

**Authors:** Zi-Ming Zhang, Hui-Jun Liu, Gang Li, Ying He, Xin Guo, Fang Zhao, Ying-Jie Luo

**Affiliations:** ^1^ Department of Medical Psychology, Tianshui Third, People’s Hospital, Tianshui, Gansu, China; ^2^ Department of Infection Management, Tianshui Third People’s Hospital, Tianshui, Gansu, China

**Keywords:** community, depression, anxiety, elderly, comprehensive psychological interventions

## Abstract

**Objective:**

To observe the intervention effect of comprehensive psychological interventions on the mental health of the elderly population.

**Methods:**

133 elderly aged 60 and above in two urban districts of Tianshui City from January 2020 to December 2020 were selected and divided into the intervention group (n=67) and the control group (n=66). The intervention group received comprehensive psychological interventions, with no intervention given to the control group. The anxiety rate, depression rate, loneliness rate and happiness rate of the two groups were collected and compared pre- and post-intervention. Self-Rating Anxiety Scale (SAS), Self-Rating Depression Scale (SDS), University of California, Los Angeles Loneliness Scale (UCLA) and Memorial University of Newfoundland Scale of Happiness (MUNSH) were used to compare the psychological status of the elderly pre- and post-intervention.

**Results:**

Differences in the inter-group main effects and time-point main effects for SAS, SDS, UCLA, and MUNSH scores of the intervention group were significant (all p<0.05). The SAS, SDS, and UCLA scores of the intervention group were higher than those of the control group after intervention. Meanwhile, the SAS and SDS scores of the intervention group were lower than those of the control group after intervention (all p<0.05). Moreover, the MUNSH score of the intervention group was higher than that of the control group at 1-year follow-up post-intervention (p<0.05). Compared with pre-intervention values, the proportions of anxiety, depression loneliness, and happiness in the intervention group were improved at 1-year follow-up post-intervention (all P<0.05).

**Conclusion:**

This study provides basis and important support for further investigations and the monitoring of health indicators in a population as fragile as the elderly. Targeted comprehensive psychological interventions can improve the negative emotions of community-dwelling elderly and maintain their physical and mental health. The “community-hospital linkage” mental health service model can improve the mental health status of community-dwelling elderly.

## 1 Introduction

With the increasingly severe trend of population aging in China, the mental health problems of the elderly have become increasingly prominent (1). Studies at home and abroad have reported that community-dwelling elderly are prone to experiencing some negative emotions due to changes in age, living status, physical health, finance, and marriage, which can in turn trigger a series of psychological problems such as loneliness, anxiety, depression, fear, and prejudice (2). Among them, the detection rates of anxiety and depression are up to 40.8% and 57.2%, respectively, which have become prevalent among the elderly in China, seriously affecting their physical and mental health and quality of life (3, 4). Moreover, it has also been found that loneliness is a key factor affecting the mental health of the elderly, and subjective well-being can also directly affect their physical and mental health (5). If not properly addressed and resolved, these negative emotions may develop into serious psychological and mental disorders while exacerbating the physical illnesses of the elderly (6). Therefore, the mental health and psychological well-being of the community-dwelling elderly cannot be overlooked. Currently, interventions for the mental health problems of the elderly are mostly carried out from the perspectives of mental health education, community activities, improving social support, and community follow-up, and there are relatively few interventions from the perspective of professional psychological treatment (7, 8). Although previous research has demonstrated the importance of community factors for a range of mental health outcomes (9). Effective interventions are still up for debate and standardized processes have yet to be developed. The existing community intervention programs still have great limitations in maintaining the long-term effect (10).

Comprehensive psychological intervention is a social psychological service model carried out in the community-hospital setting, which can improve patients’ mental health through individual psychological counseling and support, psychological coping guidance and cognitive reconstruction, group mental health education, psychological counseling, and psychological nursing care (11). This model integrates psychological counseling, mental health care, mental health education, individual and family management and other interdisciplinary, which has significant advantages (12). At present, the comprehensive psychological intervention model is widely applied in the postoperative rehabilitation of cancer patients and those with chronic diseases, without sufficient research on its application in the mental health management of community-dwelling elderly populations (13). Therefore, professional psychological treatment and counseling were utilized as part of comprehensive psychological interventions in this study to analyze their impact on anxiety, depression, loneliness, and happiness among the elderly in local communities, thereby providing a reference for improving and resolving anxiety and depression in the elderly population, mitigating their sense of loneliness, maintaining their physical and mental health, and improving their subjective well-being and quality of life.

## 2 Materials and methods

### 2.1 Study subjects

A stratified cluster random sampling method was adopted, in which 3 communities were randomly selected from the Qinzhou and Maiji districts of Tianshui City. A total of 133 community-dwelling elderly aged 60 and above in the two districts of Tianshui City were selected from January 2020 to December 2020 as the survey subjects, and a simple random generator was used for grouping, with subjects of odd and even numbers as the intervention group (n=67) and the control group (n=66), respectively. Inclusion criteria: ① Age ≥60 years; ② SDS score ≥50 or SAS score ≥50; ③ Living in the community for >1 year; ④ those without a history of cognitive impairment or mental illness; ⑤ those with clear consciousness; ⑥ those who were willing to participate in the survey. Exclusion criteria: Those with a history of cognitive impairment or mental illness and unclear consciousness who refused to sign the informed consent form. Withdrawal criteria: Participants who were unable to fully participate in the study or who were absent from the offline comprehensive psychological intervention due to the COVID-19 quarantine policy. Participants who launch the study midway for any reason.

This study was approved by the Ethics Review Committee of our hospital, and all participants were informed and offered their consent.

### 2.2 Research tools and methods

#### 2.2.1 Self-rating anxiety scale

The standard score cutoff is 50 points, with SAS standard score <50 for normal, 50-59 points for mild anxiety, 60-69 points for moderate anxiety, and ≥70 points for severe anxiety. The higher score indicates more obvious anxiety (14).

#### 2.2.2 Self-rating depression scale

It consists of 20 items, including 10 positively scored items and 10 negatively scored items. A 4-point scale was adopted to rate the frequency of depression for each item, ranging from never or rarely, infrequent, quite a lot of time, and most or all of the time. The scores of 20 items were summed to obtain a total crude score, which was multiplied by 1.25 to obtain the standardized score (take an integer). According to the Chinese normative standard, a SAS score of < 50 is considered normal, with 50-59 for mild depression, 60-69 for moderate anxiety, and ≥70 for severe anxiety. The higher indicates more obvious depression (15).

#### 2.2.3 University of California, Los Angeles Loneliness Scale (Version 3)

Complied by Russell et al. and revised and translated into Chinese by Pro. Wang Dengfeng (16) in 1995, the scale consists of 20 items, including 11 positively scored items and 9 negatively scored items. A 4-point rating scale is used to evaluate the degree of loneliness due to the discrepancy between the desired and actual levels of social interaction, with 1 point for “never”, 2 points for “rarely”, 3 points for “sometimes”, and 4 points for “always”. The total score ranges from 20-80 points, where 20-34 for low loneliness, 35-49 for moderate loneliness, 50-64 for moderately high loneliness, and 65-80 for high loneliness. The higher score indicates a stronger sense of loneliness for the individual.

The above 3 scales have been widely used in China, with high reliability and validity.

#### 2.2.4 Memorial University of Newfoundland Scale of Happiness

Complied by Kozma and Stones in 1980, the full scale includes 24 items. The revised MUNSH used in China consists of 12 positive factors (5 items for positive emotions and 7 items for negative experiences) and 12 negative factors (5 items for negative emotions and 7 items for negative experiences), with a total of 24 items in 2 dimensions. A 3-point rating scale is used, with 2 points for “yes”, 1 point for “maybe”, and 0 points for “no”. The total score = (the positive factor score - negative factor score) +24, ranging from 0 - 48 points, with ≤12 for low happiness, 13-35 for moderate happiness, and ≥36 for high happiness. The higher score suggests higher subjective well-being. With a Cronbach’s α coefficient of 0.827, this scale is applicable to assessing the happiness status of urban community-dwelling elderly populations (17).

### 2.3 Research methods

#### 2.3.1 Questionnaire investigation

The questionnaire survey was conducted by trained research staff, who used standardized instructions, question sequences, and explanations, informed each participant of the informed content and the principle of confidentiality, and distributed the questionnaire after obtaining the participant’s consent and signature. During the survey, if participants were unable to fill out the questionnaire due to age, education level, or writing difficulties, the investigators would ask the questions one by one and ask the participants to make their assessments, with their responses recorded by the investigators.

#### 2.3.2 Comprehensive psychological interventions

The comprehensive psychological interventions were formulated based on the review of relevant literature, clinical treatment experience, and the psychological problems found among the community-dwelling elderly during this survey. The intervention group consisted of qualified psychotherapists, counselors, and nursing specialists, with interventions performed 2 times per month, each session lasting at least 60 min, for a total duration of 3 months. Intervention details and methods are as follows:

1. Individual psychological counseling and support (18):
   1.1 A relatively quiet environment in the community was selected to provide individual counseling and support, generally around 60 min, and could be extended appropriately in special cases. During the psychological counseling, the professionals would remind the elderly how to use the “psychological assistance hotline” and arrange for follow-up visits.
   1.2 At the beginning of the counseling, the specialists would start with caring for the physical and mental health of the elderly, patiently interacted and communicated with them, listened to their ideas with empathy, established a good trust relationship, and provided psychological support.
   1.3 The elderly were instructed to express their inner feelings to vent out and release their negative emotions to a certain extent.
2. Guidance on psychological coping and cognitive reconstruction: Discussing the causes and roots of the psychological problems of the elderly while respecting their feelings, encouraging them to analyze the ways and attitudes for handling events, proposing problem-solving methods, guiding them to shift their perspectives through interactive exchanges, and promptly providing guidance on coping skills, teaching them some simple and easy-to-master relaxation methods, such as breathing relaxation, recalling or imagining pleasant past events, etc., thus improving their cognitive levels, enhancing their psychological resilience, rebuilding a healthy mentality and behavior, and alleviating and eliminating psychological problems to a certain extent (19).
3. Group mental health education: Community-dwelling elderly with psychological problems were organized for participation along with community staff. The professional team in this study provided group mental health counseling and explained mental and psychological health knowledge, early identification of psychological problems, coping methods, etc. to the elderly (20). Through group lectures and interactive discussions, the elderly would learn ways to maintain mental health and enhance their confidence in overcoming illnesses.
4. Mental health consultation (21): The psychological status of the elderly was monitored using the “psychological assistance hotline” to promptly conduct online follow-up visits and psychological interventions, with a total of 63 hotline follow-up visits and 56 individual psychological counseling sessions.
5. Psychological nursing care (22): During the implementation of the comprehensive psychological interventions, nursing specialists monitored the blood pressure and glucose of the community-dwelling elderly, provided health guidance and education, chatted with them, listened carefully to their troubles, identified “risk signals”, and promptly provided counseling and resolution, making it possible for the elderly to feel the psychological care, understanding, and respect from the specialists.

#### 2.3.3 The control group

Participants in the control group were given supportive treatment, including mental health knowledge pamphlets and brief mental health education (about 30minutes) after recruitment, Then, they did not receive additional intervention programs throughout the study.

### 2.4 Outcome indicators


Anxiety Rate=(Mild anxiety+moderate anxiety+severe anxiety)/total number of participants

Depression Rate=(Mild depression+moderate depression+severe depression)/total number of participants

Loneliness Rate=(Mild loneliness+moderate loneliness+severe loneliness)/total number of participats

Happiness Rate=(Moderate happiness+high happiness)/total number of participants

SAS, SDS, UCLA, and MUNSH scales were utilized to evaluate the psychological status of the elderly in the 2 groups pre-intervention and at the end of Weeks 4, 8, and 12 and one-year follow-up post-intervention.

### 2.5 Statistical methods

All survey data were entered into Excel2019 for processing, with all obtained data analyzed and processed using SPSS 25.0 statistical software. Quantitative data were statistically expressed as mean ± standard deviation (x ± s), with the t-test utilized for inter-group comparisons, and analysis of variance was adopted for comparison of data from repeated measurements. Qualitative data were expressed as cases or percentages (%), and the Chi-square test was used for inter-group comparisons. P < 0.05 was considered significantly different.

## 3 Results

### 3.1 Baseline characteristics of study subjects

The intervention group: 67 individuals, aged 60-85, with an average age of 67.99 ± 6.40 years, and a male-female ratio of 1:1.39; the majority of patients had a spouse (91.04%); for education level, the majority were illiterate or with primary school education (50.74%); for occupation, the majority were employed (61.19%); for the living situation, the majority lived with their family (79.10%); for income source, the majority relied on retirement pensions (52.24%); for community activities, the majority participated in community activities (50.75%); for mood, the majority felt happy (68.66%); and the majority suffered chronic diseases (68.66%). The control group: 66 individuals, aged 60-85, with an average age of 69.30 ± 7.87 years, and a male-female ratio of 1:1.64; the majority of patients had a spouse (84.85%); for education level, the majority were illiterate or with primary school education (51.51%); for occupation, the majority were employed (69.70%); for living situation, the majority lived with their family (81.82%); for income source, the majority relied on retirement pensions (66.67%); for community activities, the majority did not participate in community activities (53.03%); for mood, the majority felt happy (66.67%); and the majority suffered chronic diseases (62.12%).

The X^2^ or t-test suggested no significant differences (P>0.05) between the 2 groups of community-dwelling elderly in terms of gender, age, marital status, education level, pre-retirement occupation, living situation, income source, participation in community activities, mood, and presence of chronic diseases. See **Table 1**.

**Table 1:** Baseline characteristics of study subjects [n(%)/ $\bar{x}$ ± s].

Item	Intervention group (n=67) (%)	Control group (n=66) (%)	χ^2^/t	P
Gender			0.212	0.645
Male	28 (41.79)	25 (37.88)		
Female	39 (58.21)	41 (62.12)		
Age (year)	67.99 ± 6.40	69.30 ± 7.87		
Marital status				
With spouse	61 (91.04)	56 (84.85)	1.206	0.272
No spouse	6 (8.96)	10 (15.15)		
Education Level			1.270	0.530
Illiterate or primary school	34 (50.75)	34 (51.51)		
Junior high school	18 (26.87)	13 (19.70)		
High school/secondary school or above	15 (22.39)	19 (28.79)		
Pre-retirement occupation			1.063	0.303
Job	41 (61.19)	46 (69.70)		
Unemployed	26 (38.81)	20 (30.30)		
Living alone status			0.156	0.693
Living alone	14 (20.90)	12 (18.18)		
Not living alone	53 (79.10)	54 (81.82)		
Economic Source			3.288	0.193
Social Security Pensions	16 (23.88)	13 (19.70)		
Retirement pension	35 (52.24)	44 (66.67)		
Other	16 (23.88)	9 (13.63)		
Community Activity Participation			0.190	0.663
Involved	34 (50.75)	31 (46.97)		
Not involved	33 (49.25)	35 (53.03)		
Mood state			4.319	0.229
Pleasure	46 (68.66)	44 (66.67)		
General	11 (16.42)	15 (22.73)		
Sullen	10 (14.93)	5 (7.58)		
Pressure-like	0	2 (3.03)		
Chronic disease conditions			0.628	0.428
Yes	46 (68.66)	41 (62.12)		
No	21 (31.34)	25 (37.88)		

### 3.2 SAS, SDS, UCLA, and MUNSH scores of community-dwelling elderly pre- and post-intervention

There was no significant difference in pre-intervention SAS scores between the 2 groups. Analysis of variance for repeated measures indicated significant differences in main effects between the groups and main effects at time points between SAS, SDS, UCLA, and MUNSH values in the intervention group (all P < 0.05), without interactions between the 2 indicators (all P > 0.05). Meanwhile, the further simple effect analysis showed that the SAS, SDS, and UCLA scores at the end of Weeks 4, 8, and 12 and 1-year follow-up post-intervention in the intervention group were lower than those in the control group, and the differences were statistically significant (P < 0.05). In addition, the intervention group exhibited higher MUNSH scores at 1-year follow-up post-intervention than the control group, with statistically significant differences (P < 0.05), as shown in **Table 2**.

**Table 2:** Comparison of SAS, SDS, UCLA and MUNSH scores of elderly people in community before and after intervention ( $\bar{x}$ ± s).

Item	Group	Pre- intervention	Post-intervention				F/P		
			4w	8w	12w	1 year	Intergroup	Time Point	Interaction
SAS	Intervention group (n=67)	48.86 ± 8.56	45.34 ± 7.76	44.79 ± 7.44	43.30 ± 7.34	43.54 ± 7.58	5.312	6.812	1.893
	Control group (n=66)	49.06 ± 10.35	48.94 ± 9.79	48.78 ± 9.85	48.87 ± 9.95	48.58 ± 9.93	0.006	0.002	0.091
SDS	Intervention group (n=67)	48.88 ± 12.08	45.30 ± 11.07	44.07 ± 10.61	43.77 ± 10.50	43.45 ± 9.77	9.174	6.102	2.017
	Control group (n=66)	51.86 ± 13.24	50.55 ± 12.29	50.61 ± 12.25	50.70 ± 12.51	50.32 ± 12.10	<0.001	0.003	0.062
UCLA	Intervention group (n=67)	29.84 ± 9.48	29.03 ± 8.39	28.90 ± 7.92	28.58 ± 7.87	28.64 ± 8.32	5.781	1.977	1.052
	Control group (n=66)	31.88 ± 10.10	31.89 ± 10.10	31.44 ± 9.37	31.82 ± 9.98	31.86 ± 10.10	0.009	0.076	0.247
MUNSH	Intervention group (n=67)	30.01 ± 6.07	31.05 ± 4.91	31.08 ± 4.81	31.21 ± 4.76	32.02 ± 4.48	3.681	1.827	0.975
	Control group (n=66)	29.14 ± 5.51	29.38 ± 5.43	29.71 ± 5.27	29.30 ± 5.79	29.18 ± 5.48	0.032	0.078	0.352

SAS, Self-Rating Anxiety Scale; SDS, Self-Rating Depression Scale; UCLA, University of California, Los Angeles Loneliness Scale; MUNSH, Memorial University of Newfoundland Scale of Happiness.

### 3.3 Comparison of anxiety, depression, loneliness, and happiness of the elderly pre- and post-intervention

No significant differences were observed in anxiety, depression, loneliness and happiness between the intervention and control groups pre-intervention before intervention (P > 0.05). Compared with pre-intervention values, rates of anxiety (100.00% vs 46.26%), depression (100.00% vs 50.74%), and loneliness (46.26% vs 26.86%) were lower while happiness rate (53.73% vs 71.64%) was higher in the intervention group post-intervention, and the differences were statistically significant (P < 0.05). See **Table 3**.

**Table 3:** Comparison of anxiety, depression, loneliness and happiness of the elderly before and after intervention.

Item	Pre-intervention		Post-intervention(1 year)		χ^2^	P
	Intervention group(n=67)	Control group(n=66)	Intervention group(n=67)	Control group(n=66)		
Anxiety level					49.224	<0.001
No anxiety	0	0	36	3		
Mild anxiety	37	39	21	35		
Moderate anxiety	19	16	7	17		
Severe anxiety	11	11	3	11		
Anxiety rate (%)	100.00	100.00	46.26	95.45		
Depression level					72.391	<0.001
No depression	0	0	33	2		
Mild depression	48	46	23	45		
Moderate depression	12	11	7	11		
Severe depression	7	9	4	7		
Depression rate (%)	100.00	100.00	50.74	96.97		
Loneliness level					5.437	0.020
No loneliness	36	34	49	37		
Mild loneliness	19	20	11	19		
Moderate loneliness	9	10	6	8		
Severe loneliness	3	2	1	2		
Loneliness rate (%)	46.26	48.48	26.86	43.94		
Happiness level					4.594	0.032
Mild happiness	31	33	19	32		
Moderate happiness	29	27	37	27		
Severe happiness	7	6	11	7		
Happiness rate (%)	53.73	50	71.64	51.51		

Chi-square test was performed after dichotomizing variables for anxiety, depression, loneliness, and happiness. The P-value represents the comparison between the two groups 1 year after the intervention.

## 4 Discussion

Comprehensive psychological interventions refer to the approach to improving self-efficacy and awareness of illness in the elderly through multidisciplinary teamwork from multiple perspectives, domains, and directions, thereby helping the elderly better cope with negative emotions and maintain mental health. In this study, the psychological status of the elderly in the comprehensive psychological intervention group and the control group were compared, with the results showing that the SAS, SDS, and UCLA scores at the end of Weeks 4, 8, and 12, and 1-year follow-up post-intervention in the intervention group were lower than those in the control group; and the MUNSH scores at the 1-year follow-up post-intervention in the intervention group were higher than those in the control group. Compared with pre-intervention values, the intervention group exhibited lower anxiety, depression, and loneliness and improved happiness at 1-year follow-up post-intervention.

This study found that the SAS, SDS, and UCLA scores in the intervention group were lower than those in the control group at the end of Weeks 4, 8, and 12 and 1-year follow-up post-intervention, indicating that comprehensive psychological interventions improved the anxiety, depression, and loneliness of the community-dwelling elderly to varying degrees. At the same time, it also shows that the comprehensive psychological intervention adopted in this study has a long-term effect on the elderly population, which highlights the effectiveness of this intervention program. This is consistent with the findings of Hou et al. (23) that comprehensive psychological interventions were effective in reducing the occurrence of anxiety and depression. Research points out the importance of the construction of community mental health and social services, especially when inequality plays a huge role in determining the results, and need medical department outside of the service, such as the population of insufficient resources, natural disasters and epidemic popular during service (24). Specifically, as a mental health service model based on community-hospital linkage, comprehensive psychological interventions are “people-oriented”, respect the wishes and psychological needs of the elderly, and provide individual psychological counseling and support, psychological coping guidance and cognitive reconstruction, group mental health education, mental health counseling, and psychological nursing care for the elderly in the intervention group who meet the inclusion criteria (9).

Additionally, the scores of anxiety, depression, and loneliness of community-dwelling elderly in the intervention group decreased to different extents pre- and post-intervention, with an increase in their scores of subjective well-being, while the control group exhibited no significant difference in the scores of anxiety, depression, loneliness, and subjective well-being pre- and post-evaluation. This suggests that the implementation of professional comprehensive psychological interventions for community-dwelling elderly can focus on solving their psychological problems, help them establish a positive coping strategy, eliminate or mitigate anxiety and depression, alleviate loneliness, maintain mental health, thereby enabling them to maintain emotional balance while improving subjective well-being. Meta-analyses of many clinical trials support the efficacy of psychological interventions for a variety of mental health problems, such as depression, anxiety, post-traumatic stress disorder, obsessive-compulsive disorder, and more (25–27). Based on this evidence, current clinical guidelines support the use of psychological interventions in routine clinical care, as well as in primary care, such as community initiatives to develop comprehensive psychological intervention programs to meet the needs of high risk populations at the grass-roots level, especially the elderly. Our findings are consistent with the observations by Lin Mei et al. (28) that comprehensive psychological interventions could significantly reduce the occurrence of adverse emotions in the elderly.

In this study, the application of comprehensive psychological interventions significantly reduced the rate of anxiety, depression, and loneliness while improving the sense of happiness. Specifically, comprehensive psychological interventions improve the psychological resilience, self-awareness, and self-efficacy of the elderly through psychological counseling, psychological guidance, cognitive restructuring, psychological nursing care, and health education, ultimately adjusting coping strategies to alleviate and eliminate their negative emotions (29). In the meantime, Zeng et al. (30) found that patients with diabetes mellitus and hypertension exhibited significantly reduced incidence of anxiety and depression after receiving comprehensive psychological interventions, which is basically consistent with this study.

However, this study still comes with certain limitations. First, due to unavoidable social factors, including the quarantine policy, the sample size is small and limited to only 3 communities, which may lead to some bias in extrapolating the results. Additionally, it only focuses on the effect of comprehensive interventions on the mental health of the elderly, without exploring the influencing factors affecting their mental health. Future multicenter intensive studies are planned to further explore the findings of this study.

## 5 Conclusion

This study provides basis and important support for further investigations and the monitoring of health indicators in a population as fragile as the elderly. With respect to the anxiety, depression, loneliness, and subjective well-being status of the community-dwelling elderly in Tianshui City, it serves as an essential guarantee for the community-dwelling elderly to spend their twilight years peacefully by providing comprehensive psychological interventions, mental health services, and psychological care to help them live a happy life and maintain their physical and mental health. This study is designed to explore the construction of a psychological intervention model and approaches suitable for the community-dwelling elderly in Tianshui City, which is a long-term and arduous task requiring further exploration, research, and refinement in the future mental health services for the elderly.

## Data Availability

The original contributions presented in the study are included in the article/supplementary material. Further inquiries can be directed to the corresponding author.
